# Evaluation of Vancomycin Therapeutic Drug Monitoring Strategies: Pharmacokinetic, Pharmacogenetic, and Clinical Perspectives in a Prospective, Real-World Cohort Study

**DOI:** 10.1007/s00228-026-04103-w

**Published:** 2026-06-18

**Authors:** İlker Kurt, Esen Gül Koçak, Meliha Şen, Bilgül Mete, Gökhan Aygün, Özkan Özdemir, Mehmet Güven Günver, Yalım Dikmen, Ömer Fehmi Tabak, B. Sönmez Uydeş Doğan, Zeliha Pala Kara

**Affiliations:** 1 Department of Pharmacology, Faculty of Pharmacy, Istanbul University, Istanbul, Türkiye; 2 Department of Infectious Diseases and Clinical Microbiology, Faculty of Medicine, Istanbul University-Cerrahpaşa, Istanbul, Türkiye; 3 Department of Medical Biology, Faculty of Medicine, Acibadem University, Istanbul, Türkiye; 4 Department of Biostatistics, Faculty of Medicine, Istanbul University, Istanbul, Türkiye; 5 Department of Anesthesiology and Reanimation, Faculty of Medicine, Istanbul University-Cerrahpaşa, Istanbul, Türkiye

**Keywords:** Vancomycin, Therapeutic drug monitoring, Acute kidney injury, Pharmacogenetic, Pharmacokinetic

## Abstract

**Purpose:**

Vancomycin therapeutic drug monitoring is essential for optimizing efficacy and reducing the risk of vancomycin-associated acute kidney injury (VA-AKI). Although recent guidelines recommend the ratio of area under the curve over 24 h to minimum inhibitory concentration (AUC_24_/MIC)–based monitoring over trough concentration-based monitoring, differences between AUC_24_/MIC estimation methods (e.g., pharmacokinetic equations and Bayesian approaches) may influence therapeutic classification in clinical practice. In addition, pharmacogenetic factors contributing to interindividual variability in vancomycin exposure remain incompletely characterized.

**Methods:**

This prospective, single-center cohort study included adult patients who received intravenous vancomycin for at least 72 h between June 2024 and April 2025. Vancomycin trough and peak plasma concentrations were measured using an enzyme-linked immunosorbent assay. AUC_24_/MIC values were calculated using pharmacokinetic equation and Bayesian method. Agreement in therapeutic classification between trough concentration–based and AUC_24_/MIC-based monitoring was assessed. Associations between the rs2789047 genetic variant and relevant parameters were also evaluated.

**Results:**

Thirty-six patients were included, of whom 38.9% developed VA-AKI. Despite strong correlations between trough concentration and AUC_24_/MIC values (*r* = 0.84–0.87), substantial discordance in therapeutic classification was observed, with agreement rates of 63.9% for pharmacokinetic equation-based and 66.7% for Bayesian-based compared with trough concentration-based monitoring. In contrast, pharmacokinetic equation-based and Bayesian-based methods demonstrated strong concordance (86.1%). Higher trough concentrations and AUC_24_/MIC values were significantly associated with VA-AKI (*p* < 0.001). Carriers of rs2789047 A-allele exhibited higher trough concentrations and reduced elimination rates.

**Conclusion:**

Trough concentration-based monitoring frequently misclassified vancomycin exposure compared with AUC_24_/MIC–based approaches. Pharmacogenetic variability may further influence vancomycin exposure.

## Introduction

Vancomycin is a glycopeptide antibiotic effective against gram-positive organisms, including Streptococcus and Enterococcus species, as well as methicillin-resistant *Staphylococcus aureus* (MRSA). Due to its narrow therapeutic index, supratherapeutic serum concentrations may lead to significant adverse drug reactions, whereas subtherapeutic exposure can result in reduced efficacy, particularly against multidrug-resistant bacteria [1–3].

The Infectious Diseases Society of America (IDSA), the American Society of Health-System Pharmacists (ASHP), and the Society of Infectious Diseases Pharmacists (SIDP) previously recommended trough concentration-based monitoring for vancomycin [4]. However, in the updated consensus guideline published in 2020 by the IDSA, ASHP, SIDP, and the Pediatric Infectious Diseases Society (PIDS), the ratio of area under the curve over 24 h to minimum inhibitory concentration (AUC_24_/MIC) was identified as the pharmacodynamic parameter most predictive of clinical efficacy. The guideline recommends targeting an AUC_24_/MIC ratio of 400–600 (assuming an MIC of 1 mg/L) in patients with suspected or serious MRSA infections, supporting the use of AUC_24_-guided monitoring rather than trough concentration-based monitoring to improve vancomycin efficacy and reduce adverse effects such as vancomycin-associated acute kidney injury (VA-AKI). Two approaches are suggested for calculating the AUC_24_/MIC ratio: Bayesian methods and pharmacokinetic equations. Accurate estimation of the AUC_24_/MIC value requires measurement of vancomycin steady-state plasma concentrations at two distinct time points [1, 2, 5, 6].

One of the major adverse effects of vancomycin is AKI, with meta-analyses reporting a prevalence ranging from 5% to 43% [6]. In addition to vancomycin exposure, concomitant use of nephrotoxic medications is a key contributor to AKI risk. A large meta-analysis demonstrated that pharmacist-led interventions reduced VA-AKI through systematic documentation and management of concurrent nephrotoxic drugs, as well as improved renal function monitoring [7].

Pharmacogenetics refers to genetic variations that influence drug response and describes gene-drug interactions [8]. A genome-wide association study (GWAS) conducted in 489 individuals of European American ancestry receiving vancomycin therapy and identified a significant association between the rs2789047 variant located at the 6q22.31 locus and increases in serum creatinine levels during treatment (risk allele A, β= −0.06, *p* = 1.1 × 10⁻⁷) [9].

The objectives of this study were to evaluate therapeutic drug monitoring (TDM) in patients receiving vancomycin therapy using trough concentrations and the AUC_24_/MIC ratio; to determine the agreement and correlation between these two monitoring approaches; and to compare the pharmacokinetic equation-based method with the Bayesian-based method for AUC_24_/MIC calculation. In addition, this study aimed to evaluate the association of the rs2789047 genetic variant with relevant clinical and pharmacokinetic parameters.

## Materials and methods

### Study design and setting

A prospective, single-center cohort study was conducted at Istanbul University-Cerrahpaşa, Cerrahpaşa Faculty of Medicine Hospital between June 2024 and April 2025. The study was approved by the Clinical Research Ethics Committee of Istanbul University-Cerrahpaşa (Approval No. 694, dated May 14, 2024).

Inclusion criteria were as follows:

1. adult patients aged ≥ 18 years who received intermittent intravenous vancomycin infusion for more than 72 h due to suspected or confirmed infectious diseases;
2. patients with a creatinine clearance ≥ 30 mL/min;
3. patients who provided written informed consent; and
4. patients with available vancomycin plasma concentration measurements and genotyped genetic variant.

Exclusion criteria were as follows:

1. pregnant or breastfeeding women;
2. patients who required renal replacement therapy within the first 72 h of vancomycin therapy;
3. patients who received concomitant anti-MRSA therapy (e.g., linezolid, daptomycin, or teicoplanin) overlapping with vancomycin for ≥ 24 h.

### Data collection

The clinical pharmacist collected data from electronic medical records, patient files, and direct communication with the relevant physicians. A standardized patient report form was used to record demographic characteristics (weight, height, sex, and age), comorbidities, microbiological results, vancomycin dosing information, timing of vancomycin administration and blood sampling, serum creatinine measurements throughout the course of vancomycin therapy, and concomitant use of nephrotoxic agents, including intravenous contrast media, amphotericin B, loop diuretics, colistin, piperacillin/tazobactam, cisplatin, tacrolimus, cyclosporine, tenofovir disoproxil fumarate, aminoglycosides, angiotensin-converting enzyme inhibitors, angiotensin receptor blockers, nonsteroidal anti-inflammatory drugs (NSAIDs), valacyclovir, valganciclovir, acyclovir, foscarnet, and ganciclovir [9].

### Vancomycin dosing information and TDM

Vancomycin dosing information was recorded for all patients. Loading doses were not routinely used. In patients without renal impairment, vancomycin was administered at 1 g every 12 h, consistent with product labeling. In patients with renal dysfunction, maintenance dosing was adjusted according to actual body weight and creatinine clearance. Creatinine clearance (mL/min) was estimated using the Cockcroft–Gault equation.

Once steady-state conditions were achieved (after ≥ 4 consecutive doses), two vancomycin plasma concentrations were obtained for AUC_24_ estimation. Sampling was performed at steady state, with a peak concentration collected 1–2 h after completion of the infusion and a trough concentration collected 30 min before the next scheduled dose, in line with the 2020 consensus guidelines [1]. All blood samples were centrifuged at 3000 rpm for 5 min to obtain plasma. Vancomycin plasma concentrations were measured using an indirect competitive enzyme-linked immunosorbent assay (ELISA) with a commercially available kit (Creative Diagnostics, DEIANJ11; Shirley, NY, USA).

Two steady-state vancomycin plasma concentrations (peak and trough) were used for AUC_24_ estimation. Pharmacokinetic equation-based AUC_24_ calculations and parameters (elimination rate constant (Kel), volume of distribution (Vd), and elimination half-life (t1/2)) were performed using a one-compartment intermittent intravenous infusion equation implemented on the VancoPK.com website. Bayesian-based AUC_24_ calculations were conducted using the TDMx software. Model selection was performed using the “Auto Select” function after TDM data had been entered, allowing selection of the population pharmacokinetic model that best fit the individual patient data. Necessary permissions were obtained from the respective platform developers. For AUC_24_/MIC calculation, the vancomycin MIC was standardized to 1 mg/L in accordance with the 2020 consensus guideline, which defines the target AUC_24_/MIC range of 400–600 on the basis of a broth microdilution MIC of 1 mg/L. As our center performs routine susceptibility testing using the E-test method, which is not directly interchangeable with broth microdilution, isolate-specific E-test MIC values were not used in this calculation [1].

### Serum creatinine measurement and VA-AKI assessment

Daily serum creatinine levels were monitored throughout vancomycin therapy. Baseline creatinine was defined as the value measured within 48 h prior to the first vancomycin dose, and peak creatinine was defined as the highest value recorded during therapy. The percentage change in serum creatinine from baseline to peak was calculated.

VA-AKI was defined in accordance with the Kidney Disease: Improving Global Outcomes (KDIGO) criteria as any of the following: an increase in serum creatinine of ≥ 0.3 mg/dL within 48 h; an increase in serum creatinine to ≥ 1.5 times the baseline value within 7 days; or a urine output of < 0.5 mL/kg/h for at least 6 h 10.

### Concordance between AUC_24_/MIC ratio and trough concentration

The relationship between the vancomycin AUC_24_/MIC ratio and trough concentration was evaluated. AUC_24_/MIC values were calculated using both pharmacokinetic equation-based and Bayesian-based methods. Patients were categorized into three groups according to their AUC_24_/MIC ratio: <400 (subtherapeutic), 400–600 (therapeutic), and > 600 (supratherapeutic). Based on trough concentrations obtained from the same patient cohort, patients were similarly classified into three groups: <15 mg/L (subtherapeutic), 15–20 mg/L (therapeutic), and > 20 mg/L (supratherapeutic) [11]. Categorical matching was used to assess agreement in therapeutic classification between the two monitoring approaches. An agreement was defined as paired estimates falling within the same pharmacodynamic category (e.g., both below, both within, or both above the target range), whereas disagreement was defined as paired estimates classified into different categories (e.g., trough concentration within the therapeutic range but an AUC_24_/MIC ratio outside the target range).

### Comparison of AUC_24_/MIC calculation methods

The agreement and correlation between pharmacokinetic equation–based and Bayesian-based AUC_24_/MIC calculation methods were also evaluated. AUC_24_/MIC values obtained using both methods were categorized into three pharmacodynamic groups: <400 (subtherapeutic), 400–600 (therapeutic), and > 600 (supratherapeutic). Categorical matching was performed to assess agreement in therapeutic classification between the two AUC_24_/MIC calculation methods [12].

### Genotyping and single nucleotide polymorphism (SNP) analysis

Genomic deoxyribonucleic acid (DNA) was extracted from patients’ peripheral blood using the QIAamp DNA Maxi Kit (Spin Protocol) (Qiagen, Hilden, Germany) according to the manufacturer’s instructions. DNA concentration was quantified with a Qubit 3.0 Fluorometer (Life Technologies, Thermo Fisher Scientific, USA) using the Qubit dsDNA High Sensitivity Assay Kit. The genomic region containing rs2789047 was amplified by polymerase chain reaction (PCR) using gene-specific primers (Forward: 5′-TGTGGGAAGACAGGGTAGGA-3′; Reverse: 5′-GCTGACTACTAGTATTGGCCTCA-3′). PCR products were verified on a 1.5% agarose gel and purified prior to sequencing. Dideoxy sequencing (Sanger sequencing) was conducted by an accredited external laboratory (LABGEN, Acibadem University, Istanbul, Türkiye) using the ABI 3500 Genetic Analyzer platform. Raw chromatogram files were visually inspected and analyzed using Chromas and BioEdit software. Sequences were aligned against the human reference genome (GRCh38) to identify nucleotide substitutions.

### Statistical analysis

Data were analyzed using the Statistical Package for the Social Sciences (SPSS), version 30.0 (IBM SPSS Statistics, Chicago, IL, USA). The Kolmogorov–Smirnov test was used to assess the normality of data distribution. Descriptive statistics were presented as means with standard deviations for normally distributed continuous variables and as medians with interquartile ranges (IQR) for non-normally distributed continuous variables. The correlations between the AUC_24_/MIC ratios and trough concentrations were assessed using Spearman’s correlation test. Categorical variables were analyzed using Fisher’s exact test. The chi-square goodness-of-fit test was used to evaluate Hardy-Weinberg equilibrium (HWE). Student’s *t*-test was applied for comparisons of normally distributed continuous variables, whereas the Mann–Whitney *U* test was used for non-normally distributed continuous variables. All reported *p*-values were two-sided, and a *p*-value of < 0.05 was considered statistically significant.

## Results

### Patient demographics, clinical characteristics, and vancomycin therapy details

A total of 36 patients met the inclusion criteria. The majority of patients were female (52.8%). In addition to vancomycin, 52.8% of patients received one or more concomitant nephrotoxic medications, with NSAIDs being the most frequently prescribed (25%). According to the KDIGO criteria, 38.9% of patients developed VA-AKI. Vancomycin therapy was initiated for empirical treatment in 58.3% of patients and for documented infection in 41.7%; MRSA was the most frequently identified pathogen (30.6%). Patient demographics, clinical characteristics, and vancomycin therapy details are summarized in Table 1.

**Table 1:** Patient demographics, clinical characteristics, and vancomycin therapy details

Variables, *n* (%) unless specified otherwise	*n* = 36
Sex	
Female	19 (52.8)
Male	17 (47.2)
Age (IQR, years)	66 (54.75–77.5)
Body mass index (IQR, kg/m^2^)	25.5 (22.95–27.77)
Admission unit	
Intensive care unit	27 (75)
Infectious diseases unit	9 (25)
Hospital length of stay (IQR, days)	16.5 (12.25–30)
Duration of vancomycin (IQR, days)	11 (7–14)
Comorbidity status	
Yes	31 (86.1)
Number of comorbidities per patient (IQR)	2 (1–3)
Comorbidities	
Diabetes mellitus	14 (38.9)
Hypertension	9 (25)
Hypothyroidism	8 (22.2)
Others	44
Number of drugs per patient (IQR)	8 (6–9)
Use of concurrent nephrotoxic medications	19 (52.8)
Number of concurrent nephrotoxic medications per patient (IQR)	1 (0–2)
Concurrent nephrotoxic medications	
Nonsteroidal anti-inflammatory drugs (NSAIDs)	9 (25)
Piperacillin-tazobactam	6 (16.7)
Furosemide	6 (16.7)
Ramipril	2 (5.6)
Acyclovir	2 (5.6)
Colistin	1 (2.8)
Gentamicin	1 (2.8)
Amikacin	1 (2.8)
Tenofovir disoproxil fumarate	1 (2.8)
Incidence of vancomycin-associated acute kidney injury (VA-AKI)	14 (38.9)
Baseline serum creatinine (IQR, mg/dL)	0.725 (0.53–1.14)
Peak serum creatinine (IQR, mg/dL)	1.045 (0.7–1.48)
Baseline creatinine clearance (IQR, mL/min)	82.95 (55.77–135.95)
Percentage change in serum creatinine (IQR, %)	27.57 (12.93–83.36)
Vancomycin indication	
Documented infection	15 (41.7)
Empirical treatment	21 (58.3)
Culture results	
Methicillin-Resistant *Staphylococcus aureus* (MRSA)	11 (30.6)
Methicillin-Resistant Coagulase-Negative *Staphylococci* (MRCoNS)	9 (25)
*Enterococcus* spp.	1 (2.8)
*Corynebacterium* spp.	1 (2.8)

### Vancomycin serum levels and pharmacokinetic analyses

The median vancomycin trough concentration was 19.4 mg/L (16.55–27.8), and the median peak concentration was 39.5 mg/L (32.5–46.87). Vancomycin serum concentrations and pharmacokinetic parameters are presented in Table 2.

**Table 2:** Vancomycin serum levels and pharmacokinetic analyses

Parameters	Values (Median (IQR))
Trough concentration (mg/L)	19.4 (16.55–27.8)
Peak concentration (mg/L)	39.5 (32.5–46.87)
Pharmacokinetic equation-based AUC_24_/MIC	685 (590.25–865.5)
Bayesian-based AUC_24_/MIC	655 (525.25–828)
Kel (1/h)	0.048 (0.036–0.063)
Vd (L)	47 (34.5–67.25)
t1/2 (h)	14.3 (10.82–19.05)

Kel, Vd, and t1/2 were calculated using a one-compartment intermittent intravenous infusion pharmacokinetic equation based on two steady-state vancomycin plasma concentrations

### Comparison of pharmacokinetic equation-based AUC_24_/MIC monitoring and trough concentration-based monitoring

**Fig. 1:**
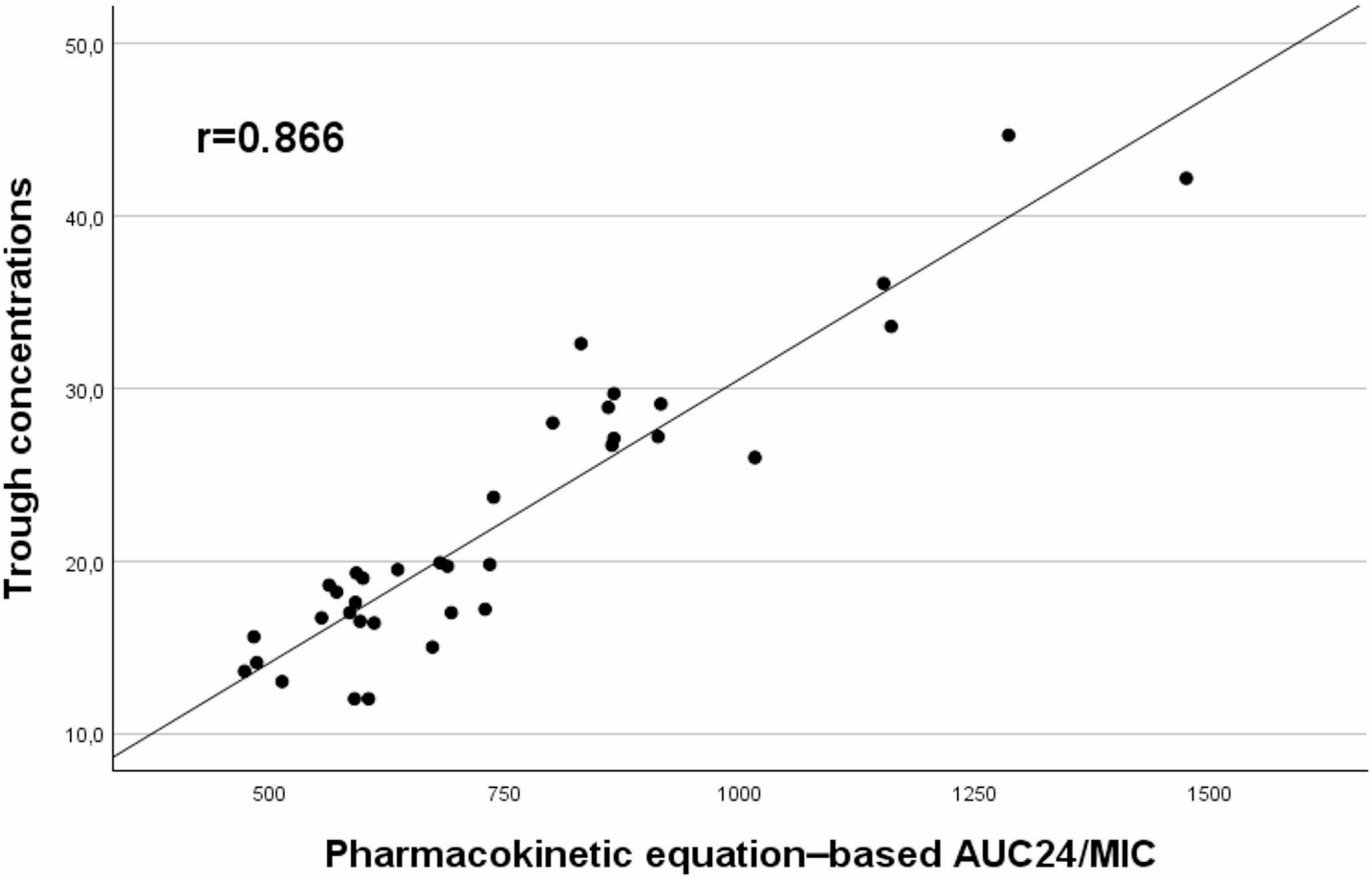
Scatter plot of pharmacokinetic equation-based AUC_24_/MIC and trough concentration-based monitoring methods

A very strong correlation was observed between pharmacokinetic equation-based AUC_24_/MIC monitoring and trough concentration-based monitoring (*r* = 0.866, *p* < 0.001). The two approaches demonstrated a 63.9% agreement in therapeutic classification (Table 3; Fig. 1).

**Table 3:** Agreement between pharmacokinetic equation-based AUC_24_/MIC and trough concentration-based monitoring methods

	Trough concentrations				
Pharmacokinetic equation-based AUC_24_/MIC		Below 15	15–20	Above 20	Total Patient Number
	Below 400	**0 (0%)**	0 (0%)	0 (0%)	0
	400–600	4 (11.1%)	**9 (25%)**	0 (0%)	13
	Above 600	2 (5.6%)	7 (19.4%)	**14 (38.9%)**	23
	**Total Patient Number**	6	16	14	**36**

therapeutic classification agreement = 23/36 = **63.9%**

### Comparison of Bayesian-based AUC_24_/MIC monitoring and trough concentration-based monitoring

**Fig. 2:**
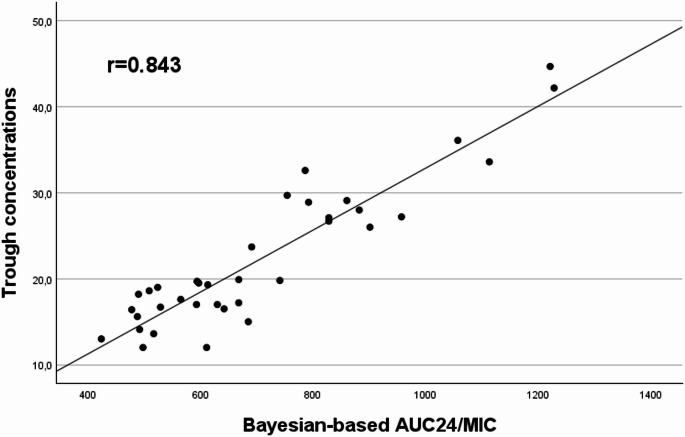
Scatter plot of Bayesian-based AUC_24_/MIC and trough level-based monitoring methods

A strong correlation was observed between trough concentration-based and Bayesian-based AUC_24_/MIC monitoring (*r* = 0.843, *p* < 0.001). The therapeutic classification agreement between the two approaches was 66.7% (Table 4; Fig. 2).

**Table 4:** Agreement between Bayesian-based AUC_24_/MIC and trough concentration-based monitoring methods

	Trough concentrations				
Bayesian-based AUC_24_/MIC		Below 15	15–20	Above 20	Total Patient Number
	Below 400	**0 (0%)**	0 (0%)	0 (0%)	0
	400–600	4 (11.1%)	**10 (27.8%)**	0 (0%)	14
	Above 600	2 (5.6%)	6 (16.7%)	**14 (38.9%)**	22
	**Total Patient Number**	6	16	14	**36**

therapeutic classification agreement = 24/36 = **66.7%**

### Comparison of pharmacokinetic equation-based AUC_24_/MIC monitoring and Bayesian-based AUC_24_/MIC monitoring

**Fig. 3:**
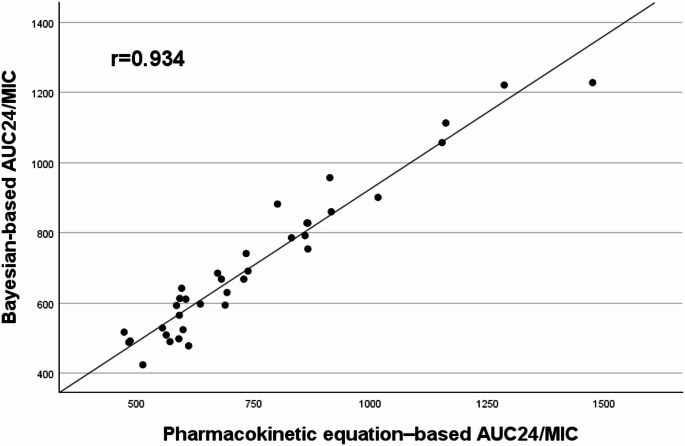
Scatter plot of pharmacokinetic equation-based AUC_24_/MIC and Bayesian-based AUC_24_/MIC monitoring methods

A very strong correlation was observed between the pharmacokinetic equation-based method and the Bayesian-based method (*r* = 0.934, *p* < 0.001), with therapeutic classification agreement of 86.1% (Table 5; Fig. 3).

**Table 5:** Agreement between pharmacokinetic equation-based AUC_24_/MIC and Bayesian-based AUC_24_/MIC monitoring methods

	Pharmacokinetic equation-based AUC_24_/MIC				
Bayesian-based AUC_24_/MIC		Below 400	400–600	Above 600	Total Patient Number
	Below 400	**0 (0%)**	0 (0%)	0 (0%)	0
	400–600	0 (0%)	**11 (30.6%)**	3 (8.3%)	14
	Above 600	0 (0%)	2 (5.6%)	**20 (55.6%)**	22
	**Total Patient Number**	0	13	23	**36**

therapeutic classification agreement = 31/36 = **86.1%**

### Clinical and pharmacokinetic determinants of VA-AKI

According to the KDIGO criteria, 38.9% of the patients developed VA-AKI. The occurrence of VA-AKI was associated with number of concurrent nephrotoxic medications, baseline creatinine clearance, percentage change in serum creatinine, vancomycin trough and peak concentrations, pharmacokinetic equation-based AUC_24_/MIC, and Bayesian-based AUC_24_/MIC values (Table 6).

**Table 6:** Incidence of VA-AKI and its association with relevant parameters

Variables, *n* (%) unless specified otherwise	VA-AKI group (*n* = 14)	Non VA-AKI group (*n* = 22)	*p*-value
Sex			
Female	8 (57.1)	11 (50)	0.742
Male	6 (42.9)	11 (50)	
Age (IQR, years)	68.5 (63.75–77)	63 (50.5–78.75)	0.346
Body mass index (IQR, kg/m^2^)	24.7 (21.5–28.55)	26.15 (24.02–27.87)	0.39
Admission unit			
Intensive care unit	13 (92.9)	14 (63.6)	0.062
Infectious diseases unit	1 (7.1)	8 (36.4)	
Hospital length of stay (IQR, days)	16.5 (13.5–33)	17 (11.75–30)	0.195
Duration of vancomycin (IQR, days)	10 (7–13.75)	11 (6.75–14)	0.961
Comorbidity status			
Yes	14 (100)	17 (77.3)	0.134
Number of comorbidities per patient (IQR)	2.5 (1–3.25)	2 (0.75–3)	0.708
Number of drugs per patient (IQR)	9 (6.75–11.5)	7 (5–8.25)	0.053
Use of concurrent nephrotoxic medications	10 (71.4)	9 (40.9)	0.097
Number of concurrent nephrotoxic medications per patient (IQR)	1.5 (0–2)	0 (0–1)	0.029*
Baseline creatinine clearance (IQR, mL/min)	67.85 (47.12–87.4)	100.4 (60.97–149.45)	0.049*
Baseline serum creatinine (IQR, mg/dL)	0.64 (0.48–1.23)	0.76 (0.56–1.1)	0.884
Percentage change in serum creatinine (IQR, %)	86.34 (45.57–148.8)	17.46 (9.14–28.29)	< 0.001**
Trough concentration (IQR, mg/L)	28.45 (23.8–32.85)	17.9 (14.77–19.72)	< 0.001**
Peak concentration (IQR, mg/L)	47.8 (42.22–62.35)	35.1 (30.67–43.07)	< 0.001**
Pharmacokinetic equation-based AUC_24_/MIC (IQR)	865 (783–1050.25)	597.5 (561–683)	< 0.001**
Bayesian-based AUC_24_/MIC (IQR)	844 (732.5–982)	579.5 (496.5–672.25)	< 0.001**
Kel (IQR, 1/h)	0.042 (0.031–0.06)	0.05 (0.043–0.064)	0.163
Vd (IQR, L)	45.5 (31.75–61.25)	52.5 (42–70)	0.363
t1/2 (IQR, h)	16.4 (11.5–22.47)	13.8 (10.75–15.9)	0.163

### Pharmacogenetic associations of vancomycin exposure and toxicity

Sanger sequencing could not be performed in one patient for technical reasons; therefore, genotype analysis of the rs2789047 variant was conducted in 35 patients. The genotype distribution was consistent with HWE, and the genotype frequencies are summarized in Table 7.

**Table 7:** Variant alleles, genotype distribution, minor allele frequencies, and HWE of the studied SNP

SNP	Variant allele	Genotype	n (%)	Minor allele frequencies	HWE	
					χ ^2^	*p-*value
rs2789047	A	CC	15 (42.86)	0.37	0.719	0.396
		AC	14 (40)			
		AA	6 (17.14)			

*SNP *Single nucleotide polymorphism, rs: reference SNP number; χ2:chi-square

Patients carrying the A allele (AC/AA genotypes) had significantly higher vancomycin trough concentration compared with those carrying the wild-type CC genotype. Kel, designated as the primary elimination metric, was significantly lower in the AC/AA group than in the CC group (Table 8).

**Table 8:** Comparison of clinical and pharmacokinetic parameters according to rs2789047 genotypes

Variables	CC (*n* = 15)	AC/AA (*n* = 20)	*p*-value	OR (95% CI)
Development of VA-AKI (n, %)			0.089	4.00 (0.86–18.64)
Yes	3 (20)	10 (50)		
No	12 (80)	10 (50)		
Percentage change in serum creatinine (IQR, %)	21.6 (10.5–65.3)	29.6 (16–86.5)	0.317	
Trough concentration (IQR, mg/L)	16.7 (14.1–19.9)	21.75 (18.7–28.67)	0.013*	
Baseline serum creatinine (IQR, mg/dL)	0.76 (0.58–1.2)	0.66 (0.38–1.14)	0.473	
Pharmacokinetic equation-based AUC_24_/MIC (IQR)	611 (555–729)	736 (593–865.5)	0.199	
Bayesian-based AUC_24_/MIC (IQR)	611 (490–685)	716 (593.25–868.5)	0.086	
Kel (IQR, 1/h)	0.057 (0.048–0.064)	0.04 (0.031–0.061)	0.012*	
t1/2 (IQR, h)	12.1 (10.8–14.4)	17.25 (11.3–22.2)	0.014*	

*OR* Odds ratio, *CI* Confidence interval

## Discussion

In this prospective cohort study, we evaluated vancomycin TDM using trough concentration-based and AUC_24_/MIC-based approaches, compared pharmacokinetic equation-based and Bayesian-based methods for AUC_24_/MIC calculation, and investigated the association between the rs2789047 genetic variant and relevant parameters. Although trough concentrations were strongly correlated with AUC_24_/MIC values, substantial discordance in therapeutic classification was observed. Higher vancomycin exposure, as reflected by elevated trough concentrations and AUC_24_/MIC values, was associated with VA-AKI. Additionally, pharmacokinetic differences observed among carriers of the rs2789047 A allele suggest a potential pharmacogenetic influence on vancomycin disposition.

These findings support the transition toward AUC_24_/MIC–guided monitoring, as recommended in the 2020 consensus guideline, by highlighting the limitations of trough concentration-based monitoring in accurately classifying vancomycin exposure. While trough concentrations were correlated with AUC_24_/MIC values, correlation alone did not ensure concordant therapeutic categorization, underscoring the importance of assessing agreement in therapeutic classification [1].

Earlier guidelines recommended trough concentrations of 15–20 mg/L as a surrogate for achieving an AUC_24_/MIC ≥ 400 in MRSA infections. However, the updated 2020 guideline advocates targeting an AUC_24_/MIC range of 400–600 (MIC = 1 mg/L), which more effectively accounts for interindividual pharmacokinetic variability and is associated with improved safety outcomes. In clinical practice, both first-order pharmacokinetic equations and Bayesian modeling are widely used to estimate AUC_24_/MIC, yet methodological differences between these approaches may still affect therapeutic classification [1, 4, 13].

Firstly, we compared pharmacokinetic equation-based AUC_24_/MIC monitoring with trough concentration-based monitoring. A very strong positive correlation was observed between the two methods (*r* = 0.866, *p* < 0.001). Previous studies comparing these approaches have reported correlation coefficients ranging from 0.51 to 0.75 [5, 13–15]. The variability in correlation strength across studies suggests that trough concentration-based monitoring provides only a partial representation of true AUC_24_/MIC exposure. This heterogeneity may be attributed to differences in patient populations, pharmacokinetic modeling approaches, methods for MIC determination, as well as the timing and accuracy of sampling strategies.

In our study, the agreement in therapeutic classification between pharmacokinetic equation-based AUC_24_/MIC monitoring and trough concentration–based monitoring was 63.9%; in other words, the two methods resulted in discordant therapeutic classifications in 36.1% of patients. This finding indicates that, despite a high correlation coefficient, clinically meaningful discrepancies may occur between the two monitoring approaches. Previous studies have reported discordance rates ranging from 21% to 75% [5, 11, 13]. Collectively, these findings indicate that high correlation coefficients do not necessarily translate into concordance in clinical classification.

Secondly, we evaluated trough concentration-based monitoring against Bayesian-based AUC_24_/MIC monitoring. A very strong correlation was observed between the two methods (*r* = 0.843; *p* < 0.001); however, the rate of therapeutic classification disagreement was 33.3%. In line with our results, a large multicenter study analyzing 26,769 dosing records reported a r² value of 0.77 between trough concentrations and Bayesian-based AUC_24_/MIC, along with a therapeutic classification disagreement rate of 34.3% [16]. Earlier studies reported an r² value of 0.51 [17], while another study identified an r value of 0.427 [18]. These findings indicate that trough concentration-based monitoring demonstrates only limited concordance with AUC_24_/MIC-based approaches whether derived from pharmacokinetic equations or Bayesian models thereby limiting the reliability of dosing decisions in clinical practice. Notably, the therapeutic classification discordance observed despite strong correlation coefficients indicates that trough levels do not provide adequate accuracy for determining individual exposure. A trough concentration within the therapeutic range may mask supratherapeutic AUC_24_/MIC exposure, whereas subtherapeutic trough levels may prompt unnecessary dose escalation in patients who already achieve adequate exposure. These differences may have important implications for both treatment failure and toxicity risk.

This study evaluated the correlation and agreement in therapeutic classification between the two AUC_24_/MIC calculation methods recommended by the 2020 vancomycin consensus guideline: pharmacokinetic equation–based and Bayesian-based approaches. A strong correlation was observed between the two methods (*r* = 0.934; *p* < 0.001), with an agreement rate of 86.1%, consistent with previous research reporting a correlation coefficient of *r* = 0.963 and an agreement rate of 87.4% [12]. Nevertheless, because the pharmacokinetic equation-based approach relies on a one-compartment structural approximation, its performance may be more limited in patients with more complex or unstable pharmacokinetic profiles. In our analysis, 3 of 23 patients (13.0%) classified as supratherapeutic according to the pharmacokinetic equation were categorized within the therapeutic range by the Bayesian model. Conversely, 2 of 13 patients (15.4%) classified as therapeutic by the pharmacokinetic equation were assessed as supratherapeutic using the Bayesian approach. In the referenced study, these proportions were reported as 21.4% and 2.23%, respectively [12]. These results indicate that when AUC_24_/MIC values are close to therapeutic thresholds, method-dependent differences may still affect classification despite strong overall agreement.

This study found a statistically significant association between the development of VA-AKI and number of concurrent nephrotoxic medications per patient (*p* = 0.029). Previous studies have demonstrated that the concomitant use of aminoglycosides, amphotericin B, acyclovir, loop diuretics, piperacillin–tazobactam, cephalosporins, and carbapenems with vancomycin substantially increases the risk of VA-AKI [19–21]. These findings highlight the need for careful clinical assessment of concurrent nephrotoxic drug use during vancomycin therapy. Owing to the limited sample size with low and heterogeneous frequency of exposure to individual nephrotoxic agents in our cohort, the individual association of each agent with VA-AKI could not be reliably assessed. Accordingly, our findings regarding nephrotoxic co-medication should be interpreted as reflecting cumulative nephrotoxic burden rather than as evidence for the effect of any specific drug combination.

Our research identified a statistically significant correlation between the development of VA-AKI and both baseline creatinine clearance (*p* = 0.049) and the percentage change in serum creatinine (*p* < 0.001). In a multicenter retrospective study, the incidence of VA-AKI was significantly higher among patients with a baseline creatinine clearance < 80 mL/min (OR = 7.73, 95% CI: 1.20–49.71) [22]. These results indicate baseline renal function and acute renal changes during treatment could be important factors in VA-AKI. This shows how important it is to thoroughly evaluate renal function before starting treatment.

Our study’s results showed significant associations between the development of VA-AKI and vancomycin trough concentrations (*p* < 0.001), peak concentrations (*p* < 0.001), and AUC_24_/MIC values calculated using both pharmacokinetic equation-based and Bayesian methods (*p* < 0.001). A systematic review identified trough concentrations > 15 µg/mL (OR: 2.10; 95% CI: 1.43–3.07) and > 20 µg/mL (OR: 2.84; 95% CI: 1.48–5.44) as significant risk factors for nephrotoxicity [19]. Another study reported that patients with trough concentrations of ≥ 20 mg/L had a markedly increased risk of VA-AKI (OR: 3.450; 95% CI: 1.146–10.390) [23]. In addition, a meta-analysis demonstrated that lower AUC_24_ exposure was associated with a significantly reduced risk of AKI compared with higher AUC_24_ levels (> 650 mg*h/L) (OR: 0.36; 95% CI: 0.23–0.56) [24]. In line with these findings, our study found that AUC_24_/MIC values were considerably higher in patients who developed VA-AKI; in other words, total vancomycin exposure is a crucial predictor of nephrotoxicity.

Because of the limited number of patients, genotype groups were analyzed under a dominant model (AC/AA vs. CC) to improve statistical stability. Importantly, the genotype distribution was consistent with HWE (χ² = 0.719, *p* = 0.396), supporting the reliability of the genotyping data. In our analysis, carriers of the rs2789047 variant exhibited significantly higher trough concentrations and lower Kel, the primary elimination metric, than non-carriers, suggesting a possible effect of this variant on vancomycin elimination. Although the percentage change in serum creatinine was higher in the variant group, the difference was not statistically significant (*p* = 0.317). In a GWAS of 489 individuals of European American ancestry receiving vancomycin, rs2789047 at chromosome 6q22.31 was significantly associated with increases in serum creatinine during treatment (β = −0.06; *p* = 1.1 × 10⁻⁷), with the A allele identified as the risk allele [9], although no significant association was reported with vancomycin trough concentrations or Kel. Taken together, these findings suggest that rs2789047 may contribute to interindividual variability in vancomycin pharmacokinetics and renal response, but larger studies are needed to clarify its clinical relevance.

## Limitations

This study has certain methodological and clinical limitations that should be considered when interpreting the findings. The single-center design, relatively small sample size (*n* = 36) and short study period may limit the applicability of our findings, and this setting may also restrict the generalizability of the results to institutions with different clinical practices and patient characteristics. In addition, AUC_24_/MIC calculations were based on a standardized MIC assumption of 1 mg/L rather than isolate-specific MIC values; therefore, absolute AUC_24_/MIC estimates and therapeutic classification may have been influenced when the true MIC differed from 1 mg/L. The limited number of events per genotype subgroup also represents an important limitation. In our study, no specific correction for multiple testing was applied; therefore, these findings should be interpreted with caution. Future studies with larger cohorts are warranted to validate these findings. Despite these limitations, the study has important strengths. To our knowledge, this is the first prospective study in Türkiye to investigate vancomycin TDM together with pharmacogenetic associations. We also believe that these findings may help guide future multi-center studies with larger sample sizes, particularly those investigating pharmacokinetic and pharmacogenetic aspects of vancomycin therapy.

## Conclusion

This study demonstrates the requirement for individualized dose management in vancomycin therapy and strongly highlights the impact of TDM on therapeutic classification. The differences observed among various monitoring approaches underline the deficiencies of using a single parameter for dose optimization. In addition, factors that directly affect treatment outcomes, such as the risk of AKI, are influenced not only by drug concentrations but also by concomitant medications and patient-specific characteristics. A comprehensive assessment of these elements, the adaptation of monitoring strategies to individual patient profiles, and the use of a multidisciplinary approach involving physicians and clinical pharmacists are essential for achieving optimal therapeutic outcomes.

## Data Availability

The data underlying this article will be shared on reasonable request to the corresponding author.

## References

[1] Rybak MJ, Le J, Lodise TP, Levine DP, Bradley JS, Liu C et al (2020) Therapeutic monitoring of vancomycin for serious methicillin-resistant *Staphylococcus aureus* infections: A revised consensus guideline and review by the American Society of Health-System Pharmacists, the Infectious Diseases Society of America, the Pediatric Infectious Diseases Society, and the Society of Infectious Diseases Pharmacists. Am J Health-System Pharm 77:835–864. 10.1093/ajhp/zxaa036 10.1093/ajhp/zxaa036 32191793

[2] Reuter SE, Stocker SL, Alffenaar JWC, Baldelli S, Cattaneo D, Jones G et al (2022) Optimal practice for vancomycin therapeutic drug monitoring: Position statement from the anti-infectives committee of the International Association of Therapeutic Drug Monitoring and Clinical Toxicology. Ther Drug Monit 44:121–132. 10.1097/FTD.0000000000000944 34882107 10.1097/FTD.0000000000000944

[3] Shenoy B, Joshi DN, Doddikoppad P (2023) Vancomycin therapeutic drug monitoring. Pediatr Infect Dis 5:17–19. 10.5005/jp-journals-10081-1387

[4] Rybak MJ, Lomaestro BM, Rotschafer JC, Moellering RC Jr, Craig WA, Billeter M et al (2009) Vancomycin therapeutic guidelines: A summary of consensus recommendations from the Infectious Diseases Society of America, the American Society of Health-System Pharmacists, and the Society of Infectious Diseases Pharmacists. Clin Infect Dis 49:325–327. 10.1086/600877 19569969 10.1086/600877

[5] Marko R, Hajjar J, Nzeribe V, Pittman M, Deslandes V, Sant N et al (2021) Therapeutic drug monitoring of vancomycin in adult patients with methicillin-resistant *Staphylococcus aureus* bacteremia or pneumonia. Can J Hosp Pharm 74. 10.4212/cjhp.v74i4.3195 10.4212/cjhp.v74i4.3195 PMC8463016 34602621

[6] Cerenzio J, Truong J (2023) Efficacy and safety of vancomycin Bayesian estimated area under the curve versus trough-based dosing. Ann Pharmacother 57:931–939. 10.1177/10600280221141402 36476049 10.1177/10600280221141402

[7] Kunming P, Xiaotian J, Qing X, Chenqi X, Xiaoqiang D, Qianzhou L (2023) Impact of pharmacist intervention in reducing vancomycin-associated acute kidney injury: a systematic review and meta-analysis. Br J Clin Pharmacol 89:526–535. 10.1111/bcp.15301 35285970 10.1111/bcp.15301

[8] Jeibouei S, Akbari ME, Kalbasi A, Aref AR, Ajoudanian M et al (2019) Personalized medicine in breast cancer: Pharmacogenomics approaches. Pharmgenomics Pers Med 12:59–73. 10.2147/PGPM.S167886 31213877 10.2147/PGPM.S167886 PMC6549747

[9] Van Driest SL, McGregor TL, Velez Edwards DR, Saville BR, Kitchner TE, Hebbring SJ et al (2015) Genome-wide association study of serum creatinine levels during vancomycin therapy. PLoS ONE 10:e0127791. 10.1371/journal.pone.0127791 26030142 10.1371/journal.pone.0127791 PMC4452656

[10] Abdelmessih E, Patel N, Vekaria J, Crovetto B, SanFilippo S, Adams C, Brunetti L (2022) Vancomycin area under the curve versus trough-only guided dosing and the risk of acute kidney injury: systematic review and meta-analysis. Pharmacotherapy 42:741–753. 10.1002/phar.2722 35869689 10.1002/phar.2722 PMC9481691

[11] Johnson-Louis KLT, Nguyen ML, Zvonar RK (2025) A comparison of vancomycin area under the curve and trough concentration in specific populations. J Pharm Pract 38(3):305-313. 10.1177/08971900241287274 39348402 10.1177/08971900241287274

[12] Olney KB, Wallace KL, Mynatt RP, Burgess DS, Grieves K, Willett A, Mani J, Flannery AH (2022) Comparison of Bayesian-derived and first-order analytic equations for calculation of vancomycin area under the curve. Pharmacotherapy 42:284–291. 10.1002/phar.2670 35134264 10.1002/phar.2670 PMC9750735

[13] Chen M, Lee C, Gnyra M, Wong M (2022) Vancomycin area under the curve/minimum inhibitory concentration and trough level concordance–evaluation on an urban health unit. Ther Adv Infect Dis 9:20499361221140368. 10.1177/20499361221140368 36465428 10.1177/20499361221140368 PMC9716452

[14] Clark L, Skrupky LP, Servais R, Brummitt CF, Dilworth TJ (2019) Examining the relationship between vancomycin area under the concentration–time curve and serum trough levels in adults with presumed or documented staphylococcal infections. Ther Drug Monit 41:483–488. 10.1097/FTD.0000000000000622 30817704 10.1097/FTD.0000000000000622

[15] Esmaeili A, Salehi M, Makhdoomi N, Ardakani YH, Rajabi M, Namazi S (2021) Evaluation of the association between trough and area under the curve to minimum inhibitory concentration ratio (AUC24/MIC) of vancomycin in infected patients with methicillin-resistant *Staphylococcus aureus*. Pharm Sci 27:201–208. 10.34172/PS.2020.70

[16] Shiau J, Roy S, Sabourenkov P, Scheetz MH (2025) Big data Bayesian truths: no vancomycin trough concentration target is sufficiently precise for safety or efficacy. Open Forum Infect Dis 12(3). 10.1093/ofid/ofaf041 40070810 10.1093/ofid/ofaf041 PMC11893978

[17] Bel Kamel A, Bourguignon L, Marcos M, Ducher M, Goutelle S (2017) Is trough concentration of vancomycin predictive of the area under the curve? A clinical study in elderly patients. Ther Drug Monit 39:83–87. 10.1097/FTD.0000000000000359 27861313 10.1097/FTD.0000000000000359

[18] Sohn YM, Park HJ, Chung JE, Huh K, Jeon K, Lee YS, Min MS (2020) Low correlation between vancomycin area under the curve over 24 hours to the minimum inhibitory concentration ratio and the trough concentration at steady state in methicillin-resistant *Staphylococcus aureus* pneumonia. J Korean Soc Health-Syst Pharm 37:408–416. 10.32429/jkshp.2020.37.4.001

[19] Kim JY, Yee J, Yoon HY, Han JM, Gwak HS (2022) Risk factors for vancomycin-associated acute kidney injury: a systematic review and meta-analysis. Br J Clin Pharmacol 88:3977–3989. 10.1111/bcp.15429 35665530 10.1111/bcp.15429

[20] Miyai T, Imai S, Kashiwagi H, Sato Y, Kadomura S, Yoshida K et al (2020) A risk prediction flowchart of vancomycin-induced acute kidney injury to use when starting vancomycin administration: A multicenter retrospective study. Antibiotics 9:920. 10.3390/antibiotics9120920 33352848 10.3390/antibiotics9120920 PMC7766575

[21] Kunming P, Can C, Zhangzhang C, Wei W, Qing X, Xiaoqiang D, Xiaoyu L, Qianzhou L (2021) Vancomycin-associated acute kidney injury: a longitudinal study in China. Front Pharmacol. 10.3389/fphar.2021.632107 33762952 10.3389/fphar.2021.632107 PMC7982802

[22] Barberán J, Mensa J, Artero A, Epelde F, Rodriguez JC, Ruiz-Morales J, Calleja JL, Guerra JM, Martínez-Gil I, Giménez MJ, Granizo JJ, Aguilar L (2019) Factors associated with development of nephrotoxicity in patients treated with vancomycin versus daptomycin for severe Gram-positive infections: a practice-based study. Rev Esp Quimioter 32:22–30 30630306 PMC6372966

[23] Pan C, Wen A, Li X, Li D, Zhang Y, Liao Y, Ren Y, Shen S (2020) Development and validation of a risk prediction model of vancomycin-associated nephrotoxicity in elderly patients: a pilot study. Clin Transl Sci 13:491–497. 10.1111/cts.12731 31785129 10.1111/cts.12731 PMC7214653

[24] Aljefri DM, Avedissian SN, Rhodes NJ, Postelnick MJ, Nguyen K, Scheetz MH (2019) Vancomycin area under the curve and acute kidney injury: a meta-analysis. Clin Infect Dis 69:1881–1887. 10.1093/cid/ciz051 30715208 10.1093/cid/ciz051 PMC6853683

